# rBCG Induces Strong Antigen-Specific T Cell Responses in Rhesus Macaques in a Prime-Boost Setting with an Adenovirus 35 Tuberculosis Vaccine Vector

**DOI:** 10.1371/journal.pone.0003790

**Published:** 2008-11-21

**Authors:** Isabelle Magalhaes, Donata R. Sizemore, Raija K. Ahmed, Stefanie Mueller, Lena Wehlin, Charles Scanga, Frank Weichold, Giulia Schirru, Maria Grazia Pau, Jaap Goudsmit, Sharon Kühlmann-Berenzon, Mats Spångberg, Jan Andersson, Hans Gaines, Rigmor Thorstensson, Yasir A. W. Skeiky, Jerry Sadoff, Markus Maeurer

**Affiliations:** 1 Microbiology, Tumor and Cell Biology Center, Karolinska Institutet, Solna, Sweden; 2 The Swedish Institute for Infectious Disease Control, Solna, Sweden; 3 Aeras Global TB Vaccine Foundation, Rockville, Maryland, United States of America; 4 Crucell Holland BV, Leiden, The Netherlands; 5 Department of Medicine, Karolinska Institutet, Karolinska University Hospital Huddinge, Stockholm, Sweden; Centre de Recherche Public-Santé, Luxembourg

## Abstract

**Background:**

BCG vaccination, combined with adenoviral-delivered boosts, represents a reasonable strategy to augment, broaden and prolong immune protection against tuberculosis (TB). We tested BCG (SSI1331) (in 6 animals, delivered intradermally) and a recombinant (rBCG) AFRO-1 expressing perfringolysin (in 6 animals) followed by two boosts (delivered intramuscullary) with non-replicating adenovirus 35 (rAd35) expressing a fusion protein composed of Ag85A, Ag85B and TB10.4, for the capacity to induce antigen-specific cellular immune responses in rhesus macaques (*Macaca mulatta*). Control animals received diluent (3 animals).

**Methods and Findings:**

Cellular immune responses were analyzed longitudinally (12 blood draws for each animal) using intracellular cytokine staining (TNF-alpha, IL-2 and IFN-gamma), T cell proliferation was measured in CD4^+^, CD8alpha/beta^+^, and CD8alpha/alpha^+^ T cell subsets and IFN-gamma production was tested in 7 day PBMC cultures (whole blood cell assay, WBA) using Ag85A, Ag85B, TB10.4 recombinant proteins, PPD or BCG as stimuli. Animals primed with AFRO-1 showed i) increased Ag85B-specific IFN-gamma production in the WBA assay (median >400 pg/ml for 6 animals) one week after the first boost with adenoviral-delivered TB-antigens as compared to animals primed with BCG (<200 pg/ml), ii) stronger T cell proliferation in the CD8alpha/alpha^+^ T cell subset (proliferative index 17%) as compared to BCG-primed animals (proliferative index 5% in CD8alpha/alpha^+^ T cells). Polyfunctional T cells, defined by IFN-gamma, TNF-alpha and IL-2 production were detected in 2/6 animals primed with AFRO-1 directed against Ag85A/b and TB10.4; 4/6 animals primed with BCG showed a Ag85A/b responses, yet only a single animal exhibited Ag85A/b and TB10.4 reactivity.

**Conclusion:**

AFRO-1 induces qualitatively and quantitatively different cellular immune responses as compared with BCG in rhesus macaques. Increased IFN-gamma-responses and antigen-specific T cell proliferation in the CD8alpha/alpha+ T cell subset represents a valuable marker for vaccine-take in BCG-based TB vaccine trials

## Introduction

Bacille Calmette-Guérin (BCG) is a safe live vaccine against *M. tuberculosis* (*Mtb*) introduced in 1921 and it is still widely used in newborns. BCG confers protection against disseminated tuberculosis (TB) during childhood, yet it fails to protect against pulmonary disease in adults. An ‘improved’ BCG, combined with boosts targeting biologically relevant *Mtb* antigens, represents a reasonable strategy to augment, broaden and prolong immune protection against TB.

An orchestrated cellular immune response of CD4^+^ [1] and CD8^+^ T cells [2], [3], [4] is required to effectively contain *Mtb* infection. Induction and expansion of antigen-specific, long-lived immune responses in CD4^+^ and CD8^+^ T cells present therefore worthy goals for vaccine development and for a rational boost strategy design. We combined a BCG prime followed by an adenovirus (Ad)-delivered Ag85A, Ag85B [5] and TB10.4 [6] boost. rAd35 was choosen due to the prevalence of Ad5-specific immune responses in Africa [7], where the TB burden is high and novel vaccination strategies are needed. For example, the seroprevalence in sub-Saharan Africa patients infected with HIV-1 of Ad5 is 90% and 20% for anti-Ad35 reactivity [8]. rAd35 induces low levels of neutralizing antibodies in non-human primates (NHPs) [9], a valuable model for preclinical TB vaccine trials: NHPs are susceptible to *Mtb* infection and develop clinical features and pathology which closely resembles TB in humans [10].

We evaluated in the current study the quantity and quality of cellular immune responses induced by BCG, or AFRO-1 (a recombinant BCG, rBCG) as the prime, followed by two subsequent adenoviral boosts. This heterologous prime-boost strategy allows the expansion of memory T cells directed specifically against Ag85A, Ag85B, and TB10.4. Expression of perfringolysin in AFRO-1 allows endosomal escape and cytosolic localization as compared to BCG. This may enhance antigen delivery and possibly a broader presentation of antigens provided by rBCG loaded onto MHC class I molecules and subsequent expansion of *Mtb*-antigen-specific CD8^+^ T cells as compared to BCG. AERAS-402 represents a recombinant, non-replicating adenovirus 35 which expresses a fusion protein of Ag85A, Ag85B and TB10.4. We examined, in longitudinally drawn blood samples, vaccine take in response to either BCG or AFRO-1 prime, followed by AERAS-402 boosts in rhesus macaques using intracellular cytokine staining (detection of TNF-α, IL-2 and IFN-γ production), T cell proliferation (in CD4^+^, CD8αβ^+^, and CD8αα^+^ T cell subsets) and IFN-γ production in 7 day T cell cultures.

## Methods

### Recombinant BCG and Adenovirus

AERAS-401, a recombinant BCG, encodes a functionally attenuated mutated perfringolysin O allele, *pfoA*G137Q which leads to perforation of the phagosomal membrane of the host cell. This enables AERAS-401 (and its passenger antigens) access to the cytosol without harmful cytotoxicity. Briefly, a mutated non-cytotoxic form of the gene encoding perfringolysin O (PfoA), was introduced into the *ureC* locus of BCG-SSI 1331 by site-directed allelic exchange mutagenesis. The AFRO-1 strain was generated by incorporating an expression plasmid encoding for three mycobacterial antigens, Ag85A (GenBank accession number P0A4V2), Ag85B (GenBank accession number P12942) and TB10.4 (GenBank accession number AF2122/97) into the Pfo-expressing BCG strain AERAS-401. Generation of AERAS-402 (rAd35-TBS), a replication deficient adenovirus serotype 35, encoding a fusion of Ag85A, Ag85B, and TB10.4, has been described previously [11].

### Animals and Immunizations

**Figure 1:**
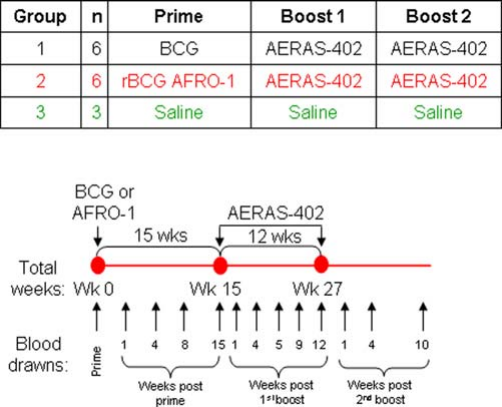
Study Timeline and Sampling Schedule. NHPs were boosted with AERAS-402 fifteen and twenty-seven weeks after the prime with BCG or AFRO-1, Animals in group 1 were primed with BCG, animals in group 2 with the recombinant BCG (AFRO-1) which combines endosomal escape, TB10.4 expression and over-expression of Ag85A and Ag85B. Animals in both groups were boosted with the non-replicating adenovirus 35 AERAS-402 which expresses the Ag85A, Ag85B and TB10.4 fusion protein. Animals in group 3 received the diluent (control group).

Female rhesus macaques (*Macaca mulatta*) of Chinese origin were 2–3 years old and housed in the Astrid Fagraeus laboratory at the Swedish Institute for Infectious Disease Control (Solna, Sweden). Housing and care procedures were in compliance with provisions and general guidelines of the Swedish Animal Welfare Agency. All procedures were approved by the Local Ethical Committee (protocol DNR238/2006-54). The study design and sampling schedule of collection of heparinized blood is shown in Figure 1. Animals in group 1 (referred as group 1 animals) were primed at week 0 with 2×10^5^ colony-forming units (CFU) with the BCG-SSI 1331 strain (reconstituted in Sauton's media), animals in group 2 (referred as group 2 animals) were primed at week 0 with 2×10^5^ CFU with AFRO-1 delivered intradermally in 0.1 ml saline. Animals in both groups were boosted twice, at 15 and 27 weeks after prime, with 2×10^11^ viral particles of AERAS-402, delivered intramuscularly in 1 mL diluent (saline). Animals in group 3 (referred as group 3 animals) received only saline.

### Antigens and Antibodies

Assessment of cellular immune responses was carried out using the Flow-cytometric Assay of Specific Cell-mediated Immune response in Activated whole blood (FASCIA) assay [12]: BCG was reconstituted from the commercially available BCG SSI1331 vaccine vial (Lot n. 106030B, Statens Serum Institut, Copenhagen, Denmark) at 2×10^6^ organisms/mL in RPMI 1640 (Gibco, Invitrogen, Stockholm, Sweden) and used at a final concentration of 2×10^5^ organisms/mL. Recombinant Ag85A, Ag85B and TB10.4 (all obtained from the Aeras Global TB Vaccine Foundation, Rockville, USA) were cloned, expressed and purified as described for Ag85B [13]. Recombinant proteins were used at final concentrations of 5 µg/mL, and purified protein derivative (PPD), obtained from Mycos Research, Loveland, USA, at 1 µg/mL in RPMI 1640. For Intracellular Cytokine Staining (ICS): Ag85A/b and TB10.4 peptide pools (15mers peptides covering the entire protein with 11 amino acids overlaps) obtained from JPT Peptide Technologies GmbH (Berlin, Germany), were used at a concentration of 1 µg/mL diluted in RPMI 1640. Staphylococcal enterotoxin A and B (SEA, and SEB; at 10 ng/mL each, Sigma-Aldrich, Stockholm, Sweden) and PMA (Phorbol Myristate Acetate) (at 25 ng/mL, Sigma-Aldrich) with ionomycin (at 1 µg/mL, Sigma-Aldrich) were used as positive controls. For the FASCIA test and ICS, the following antibodies were used to detect cell surface markers: anti-CD3 PerCP or anti-CD3 Pacific Blue (SP34-2), anti-CD4 PerCP-Cy5.5 (L200), and anti-CD8α APC-Cy7 (SK1), all obtained from BD Biosciences (Stockholm, Sweden) and anti-CD8β FITC (2ST8.5H7) was custom-conjugated at Beckman Coulter (Marseille, France). Anti-IL-2 PE (MQ1-17H12), anti-IFN-γ PE-Cy7 (B27), and anti-TNF-α APC (MAb11) were used to detected intracellular cytokines and were obtained from BD Biosciences. Note that CD8αα^+^ T cells are characterized by the cell surface detection of the CD8α chain (present as a homodimer) and the absence of CD8β chain (whereas CD8αβ^+^ T cells express the CD8α and the CD8β chain heterodimer).

### Whole Blood Antigen Stimulation Assay

40 µL of whole blood was diluted 1∶5 in RPMI 1640 L-glutamine (2 mM), penicillin (100 IU/mL) and streptomycin (10 mg/mL) (Gibco, Invitrogen) and added in duplicates to 96-well plates supplemented with Ag85A, Ag85B, TB10.4 antigens, PPD or BCG as described above. Cultures were incubated at 37°C and 5% CO_2_. After 7 days, 75 µL supernatant was removed from each duplicate well, pooled, and kept at −70°C until IFN-γ concentrations were determined using a NHP IFN-γ ELISA kit (Cell Sciences, Canton, USA).

### FASCIA

After 7 day incubation and removal of the supernatant from the WBA, cells were washed with PBS and incubated for 15 min at 4°C with the cell surface antibodies described above. Red blood cells were lysed using PharmLyse (BD Biosciences). Cell proliferation, detected by the presence of proliferative blasts, was measured by flow cytometry immediately after staining using a BD FACSAria flow cytometer (BD Biosciences). Cells were first gated on CD3 positive events, followed by a gate on resting lymphocytes and proliferative blasts based on forward scatter (FSC) vs. side scatter (SSC) parameters. The presence of CD4^+^, CD8αα^+^ and CD8αβ^+^ T cells within resting lymphocytes and proliferative blasts was assessed, and the index of proliferation (% of blasts in response to antigen stimulation - % of blasts in non-stimulated controls) was measured for each T cell compartment. Analysis was performed using FlowJo software (Tree Star Inc., Ashland, USA). Figures were generated using TIBCO Spotfire software (TIBCO Software Inc., Göteborg, Sweden).

### Intracellular Cytokine Staining

Frozen peripheral blood mononuclear cells (PBMCs) were thawed, rested overnight, and stimulated for 6 hours with peptide pools in RPMI 1640 L-glutamine (2 mM), penicillin (100 IU/mL) and streptomycin (10 mg/mL), 10% heat-inactivated FBS (Gibco, Invitrogen), in the presence of BFA (brefeldin A) (at 10 µg/mL, Sigma-Aldrich). Cells were then washed in PBS, and stained with cell surface marker antibodies as described above in the presence of the live/dead fixable dead cell marker (Aqua LIVE/DEAD; Invitrogen), for 30 min at 4°C. After washing with PBS, cells were fixed and permeabilized using the IntraPrep Fix/Perm Kit (Beckman Coulter) and incubated with antibodies specific for intracellular cytokines for 30 min at 4°C. Cells were analyzed using a BD FACSCanto flow cytometer (BD Biosciences) and analysis was performed using FlowJo software. A minimum of 10^4^ events in each individual CD4^+^, CD8αβ^+^ T cell subset and 5×10^3^ events for CD8αα^+^ were selected for analysis. Average of duplicates of the total frequency of IL-2, IFN-γ, or TNF-α producing CD4^+^, CD8αα^+^ and CD8αβ^+^ T cells stimulated with the Ag85A/b peptide pool were plotted and the proportion of the total cells expressing each of the seven possible combinations of IL-2, IFN-γ, and TNF-α from selected animals in response to: i) antigen, ii) maximal stimulation (PMA ionomycin) and iii) no stimulation (medium). The 95^th^ percentile of the percentage of cytokine-producing cells in medium control (no stimulation) was calculated. Based on these results, cutoff values for positivity above background were selected (0.15% for CD4^+^, 0.4% for CD8αα^+^, and 0.2% for CD8αβ^+^ T cells). This resulted that in the medium control determinations, 86.99% of the measurements of CD4^+^ T cells were under the cutoff, 84.55% of CD8αα^+^, and 85.37% of CD8αβ^+^ T cells, respectively. Percentage of cytokine-producing cells above these cutoffs in response to antigen stimulation were considered as positive. Note that Ag85A/b designates the peptide pool and Ag85A/B the corresponding recombinant protein as described above.

## Results

### Priming with AFRO-1 enhances IFN-γ Production in Response to Ag85A and Ag85B

**Figure 2:**
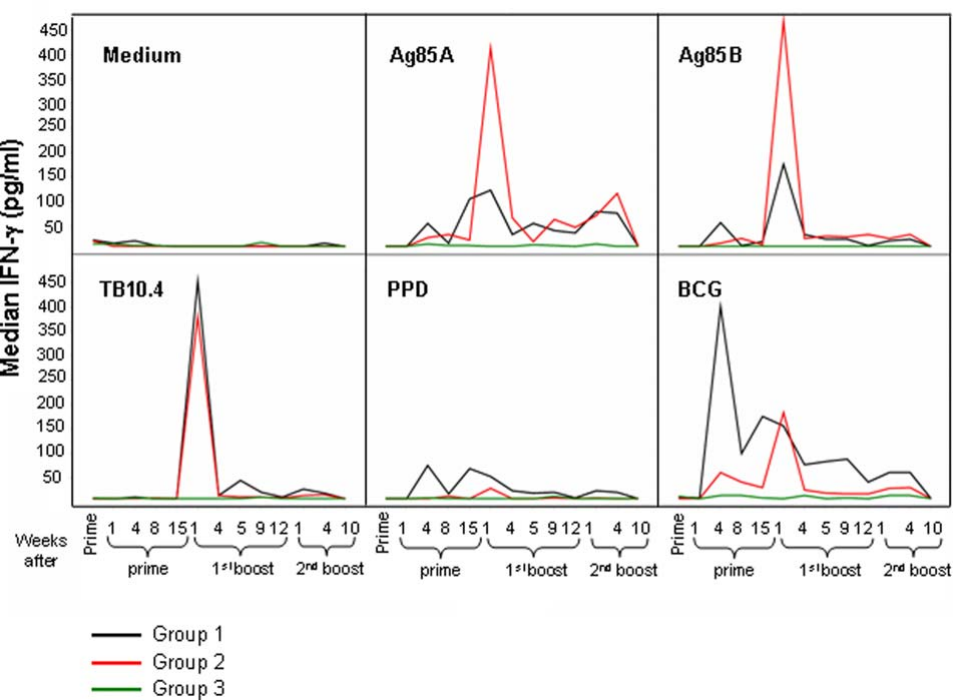
Prime with BCG or AFRO-1 induces a different IFN-γ production profile in response to *Mtb* antigen stimulation. The median of IFN-γ production (measured by ELISA) in whole blood cultures for each group in response to different *Mtb* antigen stimulation was assessed. Stronger IFN-γ production was seen in animals primed with AFRO-1 in response to Ag85A and Ag85B, as compared to animals primed with BCG one week after the first boost with AERAS-402.

NHPs were vaccinated either with BCG, or with AFRO-1 followed by two adenoviral boosts containing Ag85A/B and TB10.4 (AERAS-402) (see Figure 1). A third NHP group received only injections with diluent as a control. IFN-γ production in whole blood cultures in response to Ag85A, Ag85B, TB10.4, PPD, BCG, SEA/SEB (positive control) or no stimulation (medium, negative control) was evaluated in longitudinally sampled blood specimens (see sampling schedule detailed in Figure 1). Stimulation of whole blood with PPD or BCG induced stronger and earlier IFN-γ production (detectable four weeks after the prime) in group 1 animals as compared to group 2 animals (Figure 2). IFN-γ-induction was measured in response to each antigen-component (i.e. Ag85A, Ag85B and TB10.4) delivered as a transgene by AERAS-402: Ag85A and Ag85B stimulation induced high levels of IFN-γ production (>400 pg/mL) in group 2 animals, which peaked one week after the first boost with AERAS-402. In group 1 animals, the response to Ag85A and Ag85B, was 2 to 3-fold lower (<200 pg/mL) as compared to group 2 animals. Although an IFN-γ response was detectable as early as four weeks after the prime, the peak response was detected one week after the first boost with AERAS-402. TB10.4 stimulation of whole blood induced high levels of IFN-γ production (>400 pg/mL) in both group 1 and 2 animals one week after the first boost. Lower levels of IFN-γ production (>150 pg/ml) in response to Ag85A (for group 1 and 2 animals), and to BCG (only for group 1 animals) were observed one and four weeks after the second boost. We could not detect IFN-γ production ten weeks after the second boost in any NHP group. In summary, the BCG-prime induced IFN-γ production, defined by BCG stimulation *in vitro*, four weeks after the prime; yet it induced an overall weaker IFN-γ response directed against molecularly defined, recombinant antigens. In contrast, the AFRO-1 prime induced a delayed, but stronger IFN-γ production in response to Ag85A and Ag85B one week after the first boost with AERAS-402.

### Priming with AFRO-1 induces increased T Cell Proliferation in Response to Ag85B in CD8αα^+^ T Cells

**Figure 3:**
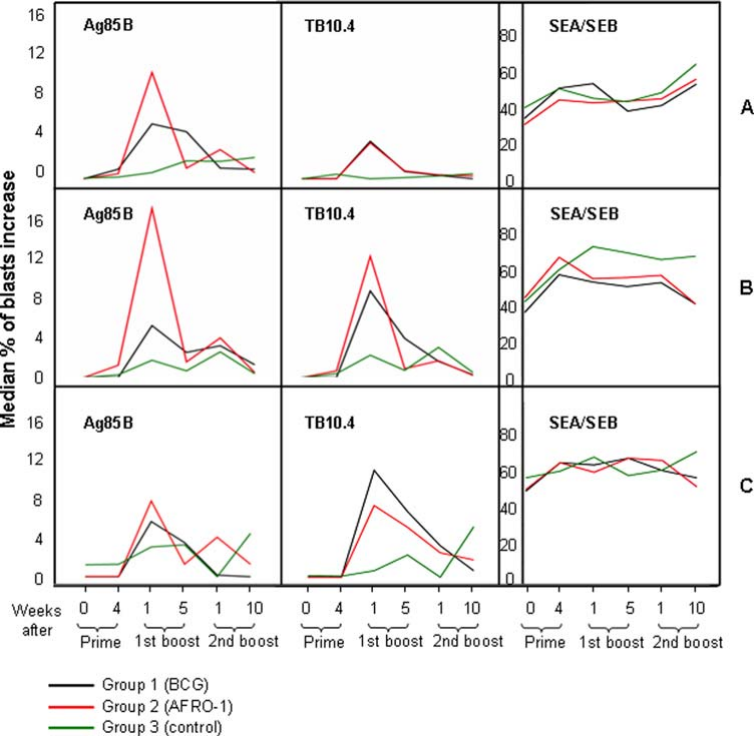
Prime with AFRO-1 induces proliferation of Ag85B-specific T cells in CD4^+^ and CD8alpha/alpha^+^ T cells. The median of the proliferation index (% of blasts in response to antigen stimulation - % of blasts in negative control) in response to *Mtb* antigens was determined by flow cytometric analysis. Differential expansion of T cell subsets was gauged by gating on T cell subsets, i.e. CD4^+^, CD8alpha/beta^+^ and CD8alpha/alpha^+^. Animals primed with AFRO-1 showed stronger proliferation in response to Ag85B stimulation within CD4^+^ T cells (A) and CD8alpha/alpha^+^ T cells (B) as compared to animals primed with BCG one week after the first boost with AERAS-402. No difference was detectable between animals primed with AFRO-1 or BCG in the CD8alpha/beta^+^ T cell compartment (C).

In order to characterize immune cell subpopulations responding to *Mtb* targets, we gauged the index of cellular proliferation within the CD4^+^, CD8αβ^+^, and CD8αα^+^ T cell subsets, since recent studies suggested that CD8αα^+^ T cells represent a biologically relevant memory T cell subset [14]. The peak of cellular proliferation in response to the test antigens occurred one week after the first boost for group 1 (BCG-primed) and group 2 (AFRO-1-primed) animals. In the CD4^+^ T cell compartment, Ag85B stimulation induced stronger stimulation in group 2 animals (proliferative index: 10%) as compared to group 1 animals (5.5%) (Figure 3A). In contrast, TB10.4 stimulation induced similar levels of proliferation (4%) in group 1 and 2 animals (Figure 3A). In the CD8αα^+^ T cell subset, Ag85B stimulation induced a stronger T cell stimulation in group 2 animals (17%) as compared to group 1 animals (5%), and TB10.4 stimulation induced comparable levels of proliferation in group 1 and group 2 animals with 9% and 12%, respectively (Figure 3B). We could not observe differences in the CD8αβ^+^ T cell subset; the proliferative index for Ag85B was 6 and 8%, and TB10.4 stimulation yielded 8 and 10% proliferation for group 1 and group 2 animals respectively (Figure 3C). No differences were observed between the different NHP groups in response to Ag85A, PPD and BCG stimulation. In summary, NHPs primed with AFRO-1 showed stronger CD4^+^ and CD8αα^+^ T cell proliferation in response to Ag85B as compared to animals primed with BCG.

### Polyfunctional Ag85A/b- and TB10.4-specific T Cells in Animals primed with AFRO-1

**Figure 4:**
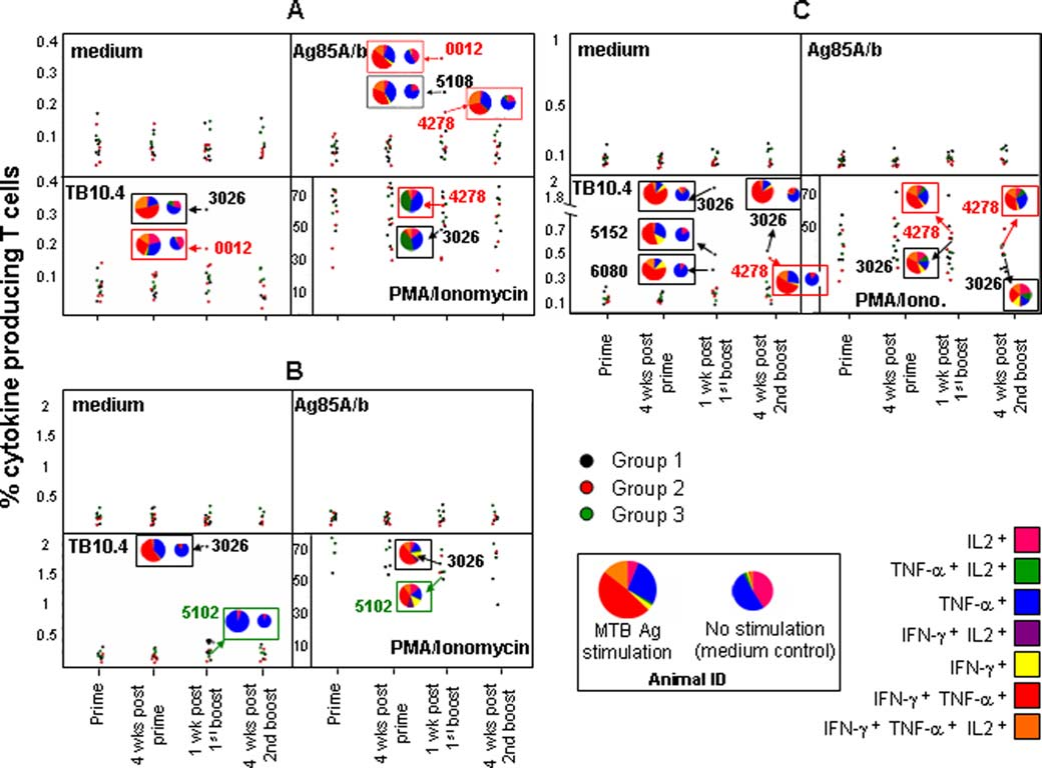
Polyfunctional Ag85A/b and TB10.4-specific T cells are present in animals primed with AFRO-1 or BCG. Production of IL-2, IFN-gamma, and TNF-alpha within CD4^+^ (A), CD8alpha/alpha^+^ (B) or CD8alpha/beta^+^ T cells (C) in response to Ag85A/b or TB10.4 peptide pool stimulation was assessed by flow cytometry. The average of duplicates of the total frequency of IL-2, IFN-gamma, or TNF-alpha producing T cells stimulated with Ag85A/b or TB10.4 peptide pools were plotted. The proportion of total T cells expressing any of the seven possible combinations of IL-2, IFN-gamma, and TNF-alpha from selected animals, either in response to antigen, maximal stimulation (PMA/ionomycin) and medium (control) are shown in pie charts. Ag85A/B and TB10.4-specific T cells were observed in 2/6 animals primed with AFRO-1, and in 4/6 animals primed with BCG one week after the first boost with AERAS-402.

Intracellular IFN-γ, TNF-α, and IL-2 production was assessed in the CD4^+^ (Figure 4A) CD8αα^+^ (Figure 4B) and CD8αβ^+^ (Figure 4C) T cell subsets using Ag85A/b or TB10.4 peptide pools. For group 2 animals, antigen-specific T cells were detected in two out of six animals. A single animal showed Ag85A/b-specific (0.35%) and TB10.4-specific (0.19%) CD4^+^ T cells, one week after the first boost (Figure 4A). In a second animal, Ag85A/b-specific (0.18%) CD4^+^ T cells could be detected one week after the first boost (Figure 4A) and TB10.4-specific (0.4%) CD8αβ^+^ T cells were detectable four weeks after the second boost (Figure 4B). For group 1 animals, antigen-specific T cells were detected in four out of six animals. One animal showed Ag85A/b-specific CD4^+^ T cells (0.25%), and two animals exhibited TB10.4-specific CD8αβ^+^ T cells (0.3 and 0.42%) one week after the first boost (Figure 4A, B). Interestingly, a single animal in group 1, displayed high numbers of TB10.4-specific CD4^+^ (0.31%), CD8αα^+^ (2%) (Figure 4C) and CD8αβ^+^ (1.9%) T cells one week after the first boost, and TB10.4-specific (0.45%) CD8αβ^+^ T cells four weeks after the second boost.

As the quality of a cellular immune response may be associated with the capacity to produce simultaneously different cytokines at the single cell level [15], the simultaneous production of IFN-γ, TNF-α, and IL-2 was measured. This allows to characterize seven distinct populations of cytokine-producing T cell subsets (see legend to Figure 4). Antigen-specific T cells displayed a unique profile of cytokine production upon *in vitro* peptide stimulation within each different T cell subset. This pattern was distinct from constitutive and maximally induced (PMA/ionomycin) cytokine production pattern in T cells. Antigen-specific CD4^+^ T cells produced TNF-α alone or in combination with IFN-γ and to a lesser extent in combination with IFN-γ and IL-2. CD8αα^+^ T cells produced predominantly TNF-α alone or in combination with IFN-γ, whereas CD8αβ^+^ T cells responses were characterized by co-production of TNF-α and IFN-γ, with detectable production of TNF-α or IFN-γ alone and simultaneous production of IFN-γ, TNF-α, and IL-2. Based on the median fluorescence intensity (MFI), CD4^+^ T cells produced six-fold more TNF-α when co-expressed with IFN-γ and ten-fold more TNF-α when co-expressed with TNF-α and IL-2 (data not shown). CD8αβ^+^ T cells produced four to five-fold more TNF-α when TNF-α was co-produced with IFN-γ alone or IFN-γ and IL-2 together. In summary, two animals primed with AFRO-1 showed polyfunctional antigen-specific T cells: one animal displayed Ag85A/b- and TB10.4-specific CD4^+^ T cells and one animal Ag85A/b-specific CD4^+^ and TB10.4-specific CD8αβ^+^ T cells. Four animals primed with BCG displayed polyfunctional antigen-specific T cells: two animals displayed only TB10.4-specific CD8αβ^+^ T cells, one animal Ag85A/b-specific CD4^+^ T cells, and one animal out the four displayed polyfunctional TB10.4-specific CD4^+^, CD8αα^+^ and CD8αβ^+^ T cells.

## Discussion

BCG vaccination prevents disseminated TB in young children, but fails to prevent adult pulmonary TB which represents the bulk of the global disease burden. New antituberculous vaccination strategies are urgently needed to improve BCG vaccination. The current study aimed to define the immunogenicity of either BCG or AFRO-1 (rBCG) priming, followed by two boosts with rAd35 AERAS-402 in rhesus macaques. Long-term (mediated by central-memory T cells) and short-term immune memory (mediated by effector-memory T cells) is most likely needed to provide effective and long-lasting protection from intracellular infections [16]. We assessed the presence of antigen-specific T cells in central memory cells (using the whole blood assay and FASCIA measuring IFN-γ production and proliferation in 7 day immune cell cultures) and in effector memory cells (measured in a six hours ICS assay). Antigen-specific responses were detected in different T cell subsets (CD4^+^, CD8αβ^+^ and CD8αα^+^). Blood obtained from animals vaccinated with BCG, but not with AFRO-1 (except in response to *in vitro* BCG stimulation) showed increased levels of IFN-γ production in response to Ag85A, Ag85B, PPD, and BCG stimulation four weeks after priming. This is consistent with antigen-specific T cell responses seen in humans after BCG vaccination [17].

IFN-γ can be produced by CD4^+^ and CD8^+^ T cells or NK cells. Therefore, we analyzed T cell proliferation in whole blood cell cultures at day 7 within the CD4^+^, CD8αβ^+^, and the CD8αα^+^ T cell compartments. The highest increase of proliferation (as compared to the control group) was observed in group 2 animals within the CD4^+^ and CD8αα^+^ T cell compartments in response to Ag85B stimulation one week after the first boost, and this was 2 to 3-fold higher than observed for group 1 animals. Stimulation with Ag85A induced higher IFN-γ production in group 2 animals but no concomitantly detectable immune cell proliferation. The discrepancy between IFN-γ production and T cell proliferation may stem from the fact that IFN-γ and proliferation is measured after 7 days of *in vitro* culture. Based on data obtained from human whole blood cultures, the level of extracellular IFN-γ remained detectable up to 7 days (whereas IL-2 protein, consumed by T cells, declines after 3 days) [18]. IFNγ-producing cells could have undergone apoptosis at day 7 and may not be detectable anymore. Alternatively, IFN-γ production may be independent of T cell proliferation: for example human HIV-1 specific CD4+ T cells produced IFN-γ in culture, but they did not proliferate [19].

Of note, Ag85B-specific production of IFN-γ, and antigen-specific proliferation peaked one week after the first boost with AERAS-402 within the CD4^+^ and CD8αα^+^ T cell compartments (see Figures 2, 3 and 4) and this pattern of reactivity was increased in animals primed with AFRO-1 as compared to animals primed with BCG. This may be due to the unique intracellular fate of AFRO-1 (rBCG) and the subsequently altered mode of antigen presentation and T cell stimulation. *Mtb* and BCG are thought to reside in the phagolysosome in macrophages, which may direct antigen presentation towards the MHC class II pathway. Interestingly *Mtb,* but not BCG, and *M. leprae* have recently been detected in the cytosol of human monocyte-derived dendritic cells and macrophages [20], a localization which may facilitate access of *Mtb* antigens to the MHC class I presentation pathway. Similarly, the endosomal escape rBCGΔ*ure*C::Hly^+^ BCG variant which expresses listeriolysin from *Listeria monocytogenes* (developed by S.H.E. Kaufman's group) [21] increases mycobacterial antigens in the cytosol of infected macrophages. Perfringolysin O expressed by AFRO-1 however, unlike the pH-dependent activity of listeriolysin which is optimal at pH≤5.5 [22], is active at an almost neutral pH of 6.5–7.0 that is typical for *Mtb* or BCG-containing phagosomal compartments; a situation which may facilitate endosomal escape.

Over-expression of Ag85B by rBCG30 was shown to induce strong cellular responses and protection against *Mtb* in guinea pigs [23], [24], but limited immunogenicity in a phase I clinical trial [25]. In contrast, AFRO-1 combines endosomal escape and over-expression of *Mtb* antigens. The novel vaccination approach presented here may therefore enable both improved access of the vaccine strain to the host cell cytoplasm and superior processing and presentation of *Mtb* antigens to CD8^+^ T cells.

We used an integrated approach in order to visualize *Mtb*-specific T cell responses directed against the vaccine-components: The whole blood cell culture assay allows measurement of central memory rather than effector memory T cells [26], [27], ICS was used to assess the presence of effector memory T cells. These cells, enriched for perforin, granzyme expression and for cytokine production are most likely key effectors in conferring immune protection [16]. Antigen-specific simultaneous production of IFN-γ, TNF-α and IL-2 was detected on the single-cell level either in CD4^+^, CD8αβ^+^, or CD8αα^+^ T cells: polyfunctional T cells may be clinically relevant and more effective as compared to single cytokine producing cells in response to *Mtb* infection [28]. For instance, IFN-γ plays a central role in the activation of infected macrophages, but recent studies suggested that IFN-γ may not correlate with protection against *Mtb* [29]. Other type-1 cytokines are instrumental in protective cellular immune responses against intracellular infections: more *Mtb* recent data consolidated the notion that polyfunctional T cells, producing TNF-α and IFN-γ with or without IL-2, play a pivotal role in protection against *Leishmania major* challenge in mice [30]. In the current study, antigen-specific T cells could only be detected in a few animals, as defined in a six hours ICS assay. It is possible that vaccination-induced antigen-specific effector memory T cells were not present in the peripheral circulation at the time of the blood draw, but rather in secondary lymph nodes [31].

Animals primed with AFRO-1 or BCG were vaccinated with AERAS-402 at 15 and 27 weeks after priming in order to boost antigen-specific memory T cells. Vaccination of mice with AERAS-402 has previously been shown to induce Ag85A, Ag85B, and TB10.4-specific T cells and to confer protection against *Mtb* [32]. We defined in the current report distinct, antigen-specific T cell subsets at different time points in the prime-boost regimen. The discrimination between CD8αα^+^ and the CD8αβ^+^ T cell subsets allowed us to demonstrate increased proliferation in response to Ag85B within the CD8αα^+^ T cell subset. This is consistent with studies obtained from healthy blood donors [33] and patients with melanoma [34]: CD8αα^+^ T cells represent a stable and distinct memory T cell population and contribute to antigen-specific memory T cell formation in mice [14] and in humans [34]. Detection of *Mtb* antigen-specific T cells in CD8αα^+^ T cells suggests that AFRO-1 activates and expands this biologically relevant memory T cell subset (see Figure 3B).

Although the current study included only a limited number of outbred animals, which did not allow to test for statistical differences, we were able to observe differences in vaccine take. The prime with AFRO-1 induces a stronger immune response as compared to BCG-prime in rhesus macaques defined by IFN-γ production and proliferation in CD8αα^+^ T cells. The latter proliferative response is absent in animals primed with BCG. The immunological readouts of immune responses in non-human primates, such as those described in the current report, allowed us to detect differences between experimental groups that reflected the response of the entire group, particularly the detection of IFN-γ and T cell proliferation in the WBA. Such assays will aid to escort future studies with novel TB vaccines in nonhuman primates and help to define clinically relevant markers of vaccine take.
